# The Relationships between Anabolic Hormones and Body Composition in Middle-Aged and Elderly Men with Prediabetes: A Cross-Sectional Study

**DOI:** 10.1155/2016/1747261

**Published:** 2016-05-03

**Authors:** Michał Rabijewski, Lucyna Papierska, Paweł Piątkiewicz

**Affiliations:** ^1^Department of Internal Diseases, Diabetology and Endocrinology, Medical University of Warsaw, Kondratowicz Street, 03-242 Warsaw, Poland; ^2^Department of Endocrinology, Medical Centre for Postgraduate Education, Marymoncka Street, 00-809 Warsaw, Poland

## Abstract

The influence of anabolic hormones and body composition in men with prediabetes (PD) is unknown. In a cross-sectional study we investigated the relationships between total testosterone (TT), calculated free testosterone (cFT), dehydroepiandrosterone sulfate (DHEAS), and insulin-like growth factor 1 (IGF-1) and body composition assessed using dual-energy X-ray absorptiometry (DXA) method in 84 patients with PD (40–80 years) and 56 men in control group. Patients with PD had lower TT, cFT, and DHEAS levels but similar IGF-1 levels in both groups. Patients with PD presented the higher total and abdominal fat as well as the lower total and abdominal lean than control (*p* < 0.02, *p* < 0.01, *p* < 0.05, and *p* < 0.02, resp.). We observed negative relationship between TT and total fat (*p* = 0.014) and positive with abdominal lean mass (*p* = 0.034), while cFT was negatively associated with abdominal (*p* = 0.02), trunk (*p* = 0.024), and leg fat (*p* = 0.037) and positively associated with total (*p* = 0.022) and trunk lean (*p* = 0.024). DHEAS were negatively associated with total fat (*p* = 0.045), and IGF-1 were positively associated with abdominal (*p* = 0.003) and leg lean (*p* = 0.015). In conclusion, the lowered anabolic hormones are involved in body composition rearrangement in men with PD. Further studies are needed to establish whether the androgen replacement therapy would be beneficial in men with PD.

## 1. Introduction

The prevalence of prediabetes (PD) in Poland is one of the highest in the world, about 16% of population is estimated to have impaired glucose tolerance (IGT), and by the year 2035, the number of people with IGT is projected to increase to about 19% [1]. PD is the condition in which the patients have slight increase in blood glucose concentrations than the normal levels but they are not said to be diabetic. The diagnostic criteria for diagnosis of PD according American Diabetes Association (ADA) are as follows: impaired fasting glucose (IFG), IGT, and/or glycated hemoglobin (HbA1c) levels from 5.7 to 6.4% [2]. PD status is considered as risk factor for the further development of diabetes mellitus type 2 (T2DM) as well as cardiovascular disease (CVD) [3].

Subnormal testosterone concentrations in men with T2DM, relative to reference ranges based on healthy young men (<12 nmol/L), are present in 25–40% of patients [4–6], but between aging, obese men, the prevalence of low T levels is around 50% [7]. Only a small proportion of these men will have classical hypogonadism because many of men with T2DM have symptoms consistent with T deficiency, but such symptoms are nonspecific and overlap with comorbidities. The clinical symptoms most commonly associated with T deficiency are low libido and erectile dysfunction, decreased muscle mass and strength, increased body fat, decreased bone mineral density and osteoporosis, depressed mood, and fatigue [8, 9]. Hypogonadism may influence not only quality of life in men, but also life span. Some observational studies show that T deficiency is associated with increased risk of all-cause and CVD-related mortality [10, 11]. These observations showed that T levels below 12 nmol/L in diabetic men may influence on the prevalence of obesity, but obesity may also result in T deficiency. There is evidence to suggest a bidirectional relationship of lowered T with increased visceral fat, which may promote a self-perpetuating cycle in diabetic men [12].

Most elderly men presenting PD also suffer from the metabolic syndrome (MetS), an insulin resistance syndrome with simultaneous occurrence of abdominal obesity, glucose metabolism disturbances, dyslipidemia, and hypertension. In our previous study we described that hypogonadism was diagnosed in 30% patients with PD in Polish population [13] but the impact of this phenomenon on body composition is unknown as well as impact of other anabolic hormones, like dehydroepiandrosterone sulfate (DHEAS) and insulin-like factor 1 (IGF-1). Only few studies have demonstrated relationships between anabolic hormones and IFG [14] or PD [15] in men but they did not assess body composition.

The aim of this study was to investigate the relationships between anabolic hormones and body composition in men with prediabetes.

## 2. Material and Methods

### 2.1. Study Population

This study was performed in Department of Internal Diseases, Diabetology and Endocrinology, Medical University of Warsaw, Poland, in male patients attending the outpatient clinic for glucose metabolism disorders. The inclusion criteria were (1) a laboratory proved PD and (2) age of 40–80 years. The exclusion criteria were as follows: (1) diabetes mellitus type 1 or 2, (2) hypercortisolism, thyroid function disturbances, (3) recent or current T replacement, androgen deprivation therapy, or any hormonal treatment, either during the study or in history, (4) recent or current treatment, which may affect body composition, for example, metformin, and (5) lack of informed written consent.

We recruited 196 consecutive patients with PD (aged between 40 and 80 years) and, as a control group, 184 men matched by age and with a fasting plasma glucose (FPG) less than 5.55 mmol/L (100 mg/dL) and HbA1c less than 5.7%. These groups were previously described in detail [13]. To final analysis we qualified patients who underwent body composition measurements (84 patients with PD and 58 control men). This study was approved by the local Research Ethics Committee and was conducted in accordance with Declaration of Helsinki; informed consent was obtained from all participants.

PD was diagnosed in patients with IFG from 100 to 125 mg/dL (5.6–6.9 mmol/L) and two-hour glucose concentration in oral glucose tolerance test (OGTT) < 140 mg/dL (<7.8 mmol/L) or in patients with IGT two-hour glucose concentration in OGTT from 140 to 200 mg/dL (7.8–11.0 mmol/L) or in patients with HbA1c from 5.7% to 6.4% [2]. The diagnosis of MetS was based on the following criteria: waist circumference (WC) ≥94 cm and any two of the following: triglycerides ≥ 150 mg/dL, HDL-cholesterol <40 mg/dL, blood pressure ≥ 130/85 mmHg, and FPG ≥ 100 mg/dL [16]. Height, weight, and waist circumference were measured and body mass index (BMI) was calculated. Obesity was defined as a body mass index (BMI) of 30 or more. Cardiovascular disease (CVD) was defined as coronary artery disease, congestive heart failure, or arrhythmia. Hypertension was considered to be present if the participant reported having received the diagnosis or if he was receiving medication for the condition.

### 2.2. Body Composition Measurement

Fat mass and lean tissue were measured using a whole-body dual-energy X-ray absorptiometry (DXA) scanner (Lunar Prodigy, GE Medical Systems, Madison, WI, USA; software version 11.4). The International Society for Clinical Densitometry recommends DXA as reference method in body composition studies and notes that this method may be useful for risk-stratification of obese patients [17]. The entire body was scanned from the top of the head. Scanned images of the whole body were subdivided into head, trunk, and left and right arm and legs. The borders between subregions were a line under the chin, a line between the humeral head and the glenoid fossa, and a line at the level of the femoral necks. The abdominal area, which is characterized by a high content of visceral fat and a low content of subcutaneous fat on MRI [18], was measured by DXA between vertebrae L2 and L4. All scans were obtained and analyzed by the same physician. DXA equipment was calibrated each day with a standardized phantom and serviced regularly. The coefficient of variation for measurements of body composition with this method is about 2%. Body fat and lean mass were expressed in kilograms. We calculated also skeletal muscle mass index (SMI (%) = total skeletal muscle mass (kg)/weight (kg) × 100). Sarcopenia was defined as an SMI of 1 SD below the sex-specific mean value for the young reference group [19].

### 2.3. Laboratory Measurements

In all patients with PD venous blood samples were obtained between 8.00 and 10.00 a.m. After centrifugation, the serum was collected and frozen at −70°C until analysis. FPG was measured with enzymatic method using BIOSEN 5040 analyzer (EKF-Diagnostic GmbH, Germany) and glycated hemoglobin (HbA1c) with HPLC method using Variant analyzer (Bio-Rad Laboratories Inc., United States). HbA1c values were expressed as % according to National Glycohemoglobin Standardization Program (NGSP). The serum levels of TT, DHEAS, estradiol (E2), and IGF-1 were measured with immunometric assays (Immulite 2000 and RIA CAC; Siemens Medical Solution, Malvern, PA, United States) and expressed in nmol/L for TT, pg/mL for E2, and ng/mL for DHEAS and IGF-1 (to convert the values for DHEAS to *μ*mol/L, multiply by 0.00271; IGF-1 to nmol/L, multiply by 0.131; and E2 to pmol/L, multiply by 3.671). To estimate the circulating fraction of FT we measured the serum level of sex hormone-binding globulin (SHBG) using an immunoassay (Diagnostic Products Corp, San Francisco, CA, United States), and SHBG was expressed in nmol/L. The serum level of calculated (cFT) expressed in nmol/L was calculated with the validated equation of Vermeulen et al. [20], according to the following formula: cFT = T − N − S + √((N + S − T)^2^ + 4NT)/2N, where T, S, and A are total testosterone (nmol/L), SHBG (nmol/L), and albumin (g/L) concentrations; N = 0.5217A + 1, using the association constants of testosterone for SHBG (10^9^ L/mol) and albumin (3.6 × 10^4^ L/mol) quoted by the authors. TT levels <12 nmol/L and cFT levels <0.250 nmol/L were taken as low.

### 2.4. Statistical Analysis

Statistical analyses were performed using the STATISTICA 9.1. data analysis software system (StatSoft, Tulsa, OK, US). Most continuous variables had a normal distribution and were expressed as a mean ± the standard deviation of the mean. The intergroup differences were tested using the *t*-test for unpaired samples. Serum DHEAS had a skewed distribution so they were log-transformed to normalize their distribution, expressed as a median with lower and upper quartiles, and the intergroup differences were tested using the *t*-test for unpaired samples for normalized values. Categorized variables were expressed as a number and a percentage, and the intergroup differences were tested using the *χ* ^2^ test. To establish correlation Spearman test was used to compare nonparametric data and Pearson test to compare parametric data. All relationships were assessed by linear univariate and multivariate regression analysis to reduce bias in a cross-sectional study. In multivariate analysis, statistical data were adjusted for age and BMI. A *p* value less than 0.05 was considered statistically significant.

## 3. Results

A total of 84 patients with PD, mean age 66.5 ± 3.8 years, and 58 control men, mean age 65.7 ± 3.8 years, were evaluated in this study. The characteristics of both groups are shown in Table 1. Patients with PD had lower TT, cFT, and DHEAS levels (*p* < 0.001, *p* < 0.005, and *p* < 0.02, resp.) and higher E2 and SHBG levels (*p* < 0.05 and *p* < 0.02, resp.). IGF-1, prolactin, and FSH did not differ between groups, while LH levels were significantly higher in patients with PD (*p* < 0.05). Patients with PD had higher HbA1c, FPG, and glucose levels in 2-hour OGTT than healthy men (*p* < 0.02, *p* < 0.001, and *p* < 0.05, resp.). Also total-cholesterol, LDL-cholesterol, and triglycerides levels were statistically significant higher in patients with PD (*p* < 0.05, *p* < 0.02, and *p* < 0.05, resp.). Systolic and diastolic blood pressure did not differ between groups; prediabetic men presented with more common hypertension, obesity, MetS, and CVD (*p* < 0.05, *p* < 0.02, *p* < 0.01, and *p* < 0.05, resp.) than control group but percentage of current smokers was similar in both groups (Table 1).

In patients with PD we have shown negative relationships between BMI and TT (*r* = −0.3594; *p* < 0.01), BMI and cFT (*r* = −0.3682, *p* < 0.01), WC and TT (*r* = −0.3476, *p* < 0.05), and WC and cFT (*r* = 0.3623; *p* < 0.02). There were also negative relationships between cFT and HbA1c (*r* = −0.3734; *p* < 0.002) and cFT and LDL-cholesterol (*r* = −0.3679, *p* < 0.02) while TT correlated significantly only with LDL-cholesterol (*r* = −0.3438, *p* < 0.05). In control group we also observed negative relationships between BMI and TT (*r* = 0.3346; *p* < 0.05), BMI and cFT (*r* = −0.3428; *p* < 0.05), and WC and cFT (*r* = −0.3478; *p* < 0.02). There was also negative relationship between cFT and HbA1c (*r* = −0.3538; *p* < 0.05) but in control group we did not observe significant relationships between androgens and lipids levels. In multivariate analysis these relationships were significant after adjustment for age and BMD.

Analysis of prediabetic patients also revealed that patients with IGT had lower TT and cFT levels than patients with IFG (*p* < 0.02). In multivariate analysis these relationships were significant after adjustment for age, BMI, and age. The difference between DHEAS and IGF-1 levels did not differ significantly among prediabetic patients with IFG or IGT (Table 2).

Standard parameters which can characterize body composition are shown in Table 1. WC were significantly higher in patients with PD when compared with control group (*p* < 0.01) while differences of weight and BMI were not statistically significant. We observed significant differences in body composition assessed by DXA methods between patients with PD and control men (Table 3). We showed the higher total and abdominal fat mass in patient with PD compared with control men (*p* < 0.02 and *p* < 0.01, resp.) as well as the lower total lean and abdominal lean mass in prediabetic patients (*p* < 0.05 and *p* < 0.02, resp.). The differences of trunk, arm, and leg fat mass as well as lean mass were not significant. SMI in both groups is also presented in Table 3. We observed slightly lower SMI in prediabetic men than in control group, but differences were not significant (mean 40.2 versus 40.5, resp.) (Table 3).

Analyses of relationships (after adjustment for age and BMI) between anabolic hormones, SHBG, HbA1c, and body composition are presented in Table 4. We have shown negative relationship between TT levels and total fat mass (*p* = 0.014) and positive with abdominal lean mass (*p* = 0.034) while cFT levels were negatively associated with abdominal (*p* = 0.02), trunk (*p* = 0.024), and leg fat (*p* = 0.037) as well as positively associated with total (*p* = 0.022) and trunk lean mass (*p* = 0.024). DHEAS levels were negatively associated only with total fat (*p* = 0.045) while IGF-1 levels were positively associated with abdominal (*p* = 0.003) and leg lean mass (*p* = 0.015). We observed also positive relationships between SHBG and abdominal fat mass (*p* = 0.024) and negative with total lean mass (*p* = 0.002). HbA1c was positively associated with total fat mass (*p* = 0.048) and negatively associated with total lean (*p* = 0.014) and abdominal lean mass (*p* = 0.034).

We observed also relationships between anabolic hormones and body composition in control group. We showed negative relationship between TT levels and total fat mass (*r* = −0.3421; *p* = 0.021) and positive with abdominal lean mass (*r* = 0.3226; *p* = 0.038) while cFT levels were negatively associated with abdominal fat (*r* = −0.3523; *p* = 0.026) as well as positively associated with total lean mass (*r* = 0.3345; *p* = 0.027). We did not observe significant relationships between DHEAS, IGF-1, and HbA1c, and parameters of body composition in control men; however, SHBG levels were negatively associated with total lean mass (*r* = −0.3453; *p* = 0.02). These relationships were still significant after adjustment for age and BMI. Analysis of relationships between SMI and anabolic hormones levels revealed that cFT levels were positively associated with SMI in prediabetic men (*r* = 0.3465; *p* = 0.02), while in control group we observed positive association SMI with IGF-1 levels (*r* = 0.3265; *p* = 0.023). These associations were significant after adjustment for BMI and age. We did not observe associations with DHEAS, TT, SHBG, and HbA1c in both groups.

## 4. Discussion

In this study we investigated the relationships between anabolic hormones and body composition in 84 men with PD from 40 to 80 years old and in 58 control aged-matched men. To achieve the goals, we have used dual-energy X-ray absorptiometry (DXA) method. In our opinion, there are two major findings arising from the present study. Firstly, patients with PD had lower TT, cFT, and DHEAS and higher E2 and SHBG levels than those observed in control men but IGF-1 levels did not differ between groups. In men with PD we observed negative relationships between BMI, WC, and TT as well as cFT levels, but these parameters were not correlated with DHEA and IGF-1 levels. These results are consistent with our previous observations [13] that, not only in men with T2DM but also among prediabetic man, the lower anabolic hormones than in healthy-peers, especially lower T levels, may by associated with glucose metabolism disorders. It seemed to us also clear that lower anabolic hormones may adversely affect body composition.

Secondly, we showed the higher total and abdominal fat mass in patient with PD compared with control group as well as the lower total lean and abdominal lean mass. We have also observed negative relationship between TT levels and total fat mass and positive with abdominal lean mass while cFT levels were negatively associated with abdominal, trunk, and leg fat mass and positively associated with total and trunk lean mass. In contrast, DHEA levels were negatively associated only with total fat while IGF-1 levels were positively associated with abdominal and leg lean mass. In our cohort SHBG showed positive correlation with abdominal fat mass and negative with total lean mass, while HbA1c was positively associated with total fat mass and negatively associated with total lean and abdominal lean mas. These results suggest that anabolic hormones have probably significant influence on body composition in patients with PD but in various regions of the body these effects are not homogenic. Our observations allow us to conclude that lower T most affects the abdominal obesity but DHEA and IGF-1 also are involved in body composition rearrangement in patients with PD. Generally, it should be noted that patients with lower levels of TT, cFT, DHEAS, and IGF-1 have a tendency to obesity and reduced muscle mass. However, it must be pointed that, in our cohort, skeletal muscle mass index (SMI), which reflects the sarcopenia, the clinical status as well as prediabetes associated with insulin resistance [21], was only slightly lower in patients with PD. It seems, therefore, that in prediabetic men lowered anabolic hormones are associated mainly with an increase of fat mass.

The prevalence of PD in Poland is one of the highest in the world [1], so any consequences of PD are a serious public health problem. We have shown previously that low T was diagnosed in 30% of patients with PD and only in 14% of control group. We also observed that low TT levels in these patients were associated with clinical signs and symptoms of hypogonadism, erectile dysfunctions, lower urinary tract symptoms, depression, and a significant deterioration in the quality of life [22, 23].

Our knowledge about the influence of anabolic hormones, especially DHEAS and IGF-1, on metabolism and body composition in patients with PD is limited. Only few studies have demonstrated relationships between androgens and PD in men. Ho et al. [15] demonstrated that PD was associated with an increased risk of subnormal TT levels compared to healthy individuals (age-adjusted OR = 1.87; 95% CI), still significant after adjusting for age, BMI, and MetS. In MEST study Colangelo et al. [14] showed that IFG was associated inversely with TT, DHEAS, and E2, and these correlations were still significant after adjustment for age and BMI. Corona et al. [24] in men with sexual dysfunction showed that 19% of them were classified as IFG and these men more often had severe erectile dysfunction or hypogonadism when compared with normoglycemic men. The relationships between another anabolic hormones and glucose metabolism disturbances have been limited. Colao et al. [25] showed that IGF1 levels in the low-normal range are associated with IFG in men, and Kameda et al. [26] observed association of low DHEAS levels with the progression of PD to T2DM in men. These results have suggested that DHEAS and IGF-1 also play an important role in glucose metabolism, but their influence on body composition in men with PD is still unknown.

Because there are no studies in men with PD, our knowledge about relationships between anabolic hormones and body composition is partially based on studies conducted in men with T2DM. In our cohort we previously showed that TT levels below 12 nmol/L were observed in 30% of prediabetic men in contrast to 14% in control group [13], while in current study we observed that androgens were negatively associated with fat mass and positively with lean body mass, independent of age and BMI. These results are consistent with data suggesting that obesity is a major correlate of lowered TT in men with glucose metabolism disorders [7]. It also demonstrates that adiposity is a major determinant of lowered TT, irrespective of whether men have T2DM or “only” PD. Whether lowered androgens are a cause or consequence of obesity cannot be answered on the basis of our studies but current evidence suggests that this relationship is bidirectional: on the one hand, lower T leads to metabolically unfavorable changes in body composition, like decreasing of lean body mass and increasing of fat mass, with associated increases in insulin resistance [27]. On the other hand, weight gain and development of T2DM contribute to the age-related decline in TT levels [28]. However, weight loss increases TT levels, suggesting that this phenomenon is functional, and to a reversible degree [7].

The possible pathophysiological mechanism between PD and lowered T is probably multifactorial and represented mainly by visceral adiposity and insulin resistance. Decreased TT levels in men are associated with insulin resistance and reduced insulin sensitivity [29] and have been found to predict insulin resistance, obesity, and T2DM [30]. Testosterone can be converted to estradiol in the adipose tissue and it has been suggested that excessive estrogens synthesis in the obese men may suppress secretion of T [31]. In our study estradiol levels were higher in men with PD than in control group. The studies in animals and in men after androgen deprivation therapy which suppress functions of androgen/androgen receptor (AR) signaling revealed significant metabolic disorders like T2DM and MetS which can be associated with tissue-specific AR signaling that is involved in regulation of metabolism [32]. Recent studies also shown that nucleotide polymorphism of the endothelial nitric oxide synthase (eNOS) gene may be an independent risk factor for T2DM and insulin resistance in men with low T [31]. Diabetic men with lowered T are significantly more likely to be obese or insulin resistant; however, the inverse association of low T with MetS or T2DM is less consistent for cFT compared with TT. This is because of the confounding effects of SHBG, itself being a strong associate of insulin resistance [33], and because SHBG but not T was inversely associated with worse glycaemic control [7, 12]. In our cohort we also demonstrated the higher SHBG levels in prediabetic patients, positive relationships between SHBG and abdominal fat mass, and negative with total lean mass. SHBG probably may have biological actions beyond serving as a carrier protein for and regulator of circulating sex steroids [33].

Importantly, in our cohort about 15% men had a TT of <8 nmol/L, a threshold level that characterizes men with severe late onset hypogonadism and is required for T replacement therapy [34], but reduction in TT levels below 12 nmol/L was observed in about 25% patients. Whether these moderate reductions in T have implications for the general health remains debated. Evidence derived from clinical studies supports the use of T replacement in hypogonadal patients, although the benefit-risk ratio is uncertain in advanced age. Replacement therapy in diabetic men with lowered T may improve insulin sensitivity, glycemic control, cardiovascular risk factors, and diabetic complications [35–38] but long-term influence of T in men with hypogonadism and PD is still unknown.

It should be noted that in our study PD was diagnosed also in patients with HbA1c from 5.7 to 6.4% [2]. Currently, an intermediate HbA1c range is not considered as PD by the World Health Organization; however, ADA definition probably gives wider range of subjects at the risk group of T2DM development, and HbA1c of 5.7–6.4% detects, in part, different individuals with intermediate hyperglycemia compared with IFG and IGT [37]. Some of the issues can be cited as weaknesses in our data set. The hormones measurements were not repeated in our sample set. It is also important to remember that we focused only on associations between body composition and hormones in men with PD. Our model in no way established a causal link between anabolic hormones levels and PD as well as body composition. These conditions might simply overlap and they have probably separate pathophysiologic pathways. We must also note that muscle is a primary target organ for insulin action, but in our study we did not measure insulin concentration, and that is why we did not evaluate the relationships between insulin and anabolic hormones, and in our cross-sectional study we did not evaluate the potential impact of exercise and physical activity on the body composition.

In conclusion, anabolic hormones are involved in body composition rearrangement in male patients with PD. Hormonal determinants of this phenomenon are different in various body areas. Further studies are needed to establish whether the androgen replacement therapy would be beneficial in men with PD.

## Figures and Tables

**Table 1:** Characteristics of men with PD and control group.

Parameter	Prediabetes number 84	Control number 58	*p* ^*∗*^
Age (years)	66.5 ± 3.8	65.7 ± 3.8	—
TT (nmol/L)	11.24 ± 1.81	16.23 ± 1.6	0.001
cFT (nmol/L)	0.331 ± 0.08	0.387 ± 0.07	0.005
DHEAS (ng/mL)	542 (186–867)	669 (229–943)	0.02
IGF-1, (ng/mL)	87.3 ± 43.3	94.3 ± 41.9	—
LH (IU/L)	6.7 ± 0.9	4.4 ± 1.2	0.05
FSH (IU/L)	8.2 ± 1.1	7.9 ± 1.3	—
Prolactin (ng/mL)	11 ± 3.8	12 ± 3.7	—
Estradiol (pg/mL)	31.9 ± 8.2	28.6 ± 7.8	0.05
SHBG (nmol/L)	31.8 ± 3.2	27.7 ± 3.4	0.02
Weight (kg)	86.2 ± 7.45	84.8 ± 6.75	—
BMI (kg/m^2^)	28.6 ± 1.2	27.8 ± 0.9	—
WC (cm)	111 (103–115)	98 (94–104)	0.01
HbA1c (%)	6.2 ± 0.9	5.2 ± 1.2	0.02
FPG (mg/dL)	116 ± 6.6	92 ± 4.6	0.001
Glucose in OGTT (mg/dL)	148 ± 6.4	117 ± 6.6	0.05
SBP (mmHg)	146 ± 17.2	139 ± 17.3	—
DBP (mmHg)	94 ± 12.4	90 ± 8.7	—
Cholesterol (mg/dL)	225 ± 18	202 ± 17	0.05
Triglycerides (mg/dL)	162 ± 13	151 ± 14	0.05
HDL-cholesterol (mg/dL)	36 ± 4.6	39 ± 4.2	—
LDL-cholesterol (mg/dL)	146 ± 12	132 ± 13	0.02

Comorbidities			
Obesity, %; (number)	71 (60)	57 (33)	0.02
Current smoker, %, (number)	32 (27)	33 (19)	NS
Hypertension, %, (number)	51 (43)	38 (22)	0.05
MetS, %, (number)	83 (70)	67 (39)	0.01
CVD, %, (number)	26 (22)	19 (11)	0.05

Data are presented as a mean ± standard deviation of the mean, a median (with lower and upper quartiles), or number (percentage), where appropriate. ^*∗*^ *p* shows differences between men with PD and control group. BMI: body mass index, WC: waist circumference, TT: total testosterone, cFT: calculated free testosterone, LH: luteinizing hormone, DHEAS: dehydroepiandrosterone sulfate, SHBG: sex hormone binding globulin, E2: estradiol, IGF-1: insulin-like growth factor 1, FPG: fasting plasma glucose, OGTT: oral glucose tolerance test, HbA1c: glycated hemoglobin, MetS: metabolic syndrome, CVD: cardiovascular disease, SBP: systolic blood pressure, and DBP: diastolic blood pressure.

**Table 2:** Anabolic hormones concentrations in patients with PD after dividing according glycemic control disorders.

Patients with prediabetes				
Parameter	All (number 84)	IFG (number 48)	IGT (number 36)	*p* ^*∗*^
TT (nmol/L)	11.24 ± 1.81	12.45 ± 1.79	10.12 ± 1.93	0.02
cFT (nmol/L)	0.331 ± 0.08	0.349 ± 0.09	0.314 ± 0.09	0.03
DHEAS (ng/mL)	542 (186–867)	545 (198–867)	539 (186–849)	—
IGF-1 (ng/mL)	87.3 ± 43.3	89.8 42.2	86.1 45.1	—
SHBG (nmol/L)	31.8 ± 3.2	31.6 ± 3.1	32.3 ± 3.4	—

^*∗*^ *p* shows differences between patients with IFG and IGT among all prediabetic patients, TT: total testosterone, cFT: calculated free testosterone, DHEAS: dehydroepiandrosterone sulfate, IGF-1: insulin-like growth factor 1, and SHBG: sex hormone binding globulin.

**Table 3:** Body composition and SMI assessed by DXA methods among patients with PD and control group.

Body composition			
Parameter	Prediabetes number 84	Control number 58	*p* ^*∗*^
Total fat (kg)	28.71 ± 2.47	26.47 ± 2.35	0.02
Abdominal fat (kg)	3.23 ± 0.23	2.48 ± 0.28	0.01
Trunk fat (kg)	15.22 ± 1.35	14.35 ± 1.41	—
Arm fat (kg)	2.68 ± 0.45	2.54 ± 0.39	—
Leg fat (kg)	7.75 ± 1.23	7.35 ± 1.35	—
Total lean (kg)	54.13 ± 3.67	56.34 ± 3.48	0.05
Abdominal lean (kg)	3.57 (3.48–3.66)	4.12 (3.98–4.27)	0.02
Trunk lean (kg)	28.23 ± 3.75	29.48 ± 3.66	—
Arm lean (kg)	6.29 ± 0.85	6.66 ± 0.91	—
Leg lean (kg)	15.65 ± 1.75	16.77 ± 2.93	—
SMI (%)	40.2 (37.6–42.8)	40.5 (37.8–43.0)	NS

Data are presented as a mean ± standard deviation of the mean or as median (with lower and upper quartiles), depending on the normality of data distribution; ^*∗*^ *p* shows differences between patients with PD and control group. DXA: dual energy X-ray absorptiometry and SMI (%): total skeletal muscle mass.

**Table 4:** Pearson's coefficients (age- and BMI-adjusted) for correlations between body composition parameters, anabolic hormones levels, SHBG, and HbA1c in patients with PD (no. 84).

Parameter	TT	cFT	DHEAS	IGF-1	SHBG	HbA1c
Total fat	*r* = −0.43	*r* = −0.06	*r* = −0.22	*r* = −0.12	*r* = 0.23	*r* = 0.47
	*p* = 0.014		*p* = 0.045			*p* = 0.048

Abdominal fat	*r* = −0.28	*r* = −0.38	*r* = −0.19	*r* = −0.16	*r* = 0.36	*r* = 0.01
		*p* = 0.02			*p* = 0.024	

Trunk fat	*r* = −0.04	*r* = −0.37	*r* = −0.21	*r* = −0.09	*r* = 0.31	*r* = 0.36
		*p* = 0.024				

Arm fat	*r* = −0.21	*r* = −0.18	*r* = −0.07	*r* = −0.24	*r* = 0.17	*r* = 0.10

Leg fat	*r* = −0.09	*r* = −0.34	*r* = −0.17	*r* = −0.06	*r* = 0.28	*r* = 0.04
		*p* = 0.037				

Total lean	*r* = 0.11	*r* = 0.38	*r* = 0.02	*r* = 0.12	*r* = −0.45	*r* = −0.34
		*p* = 0.022			*p* = 0.002	*p* = 0.014

Abdominal lean	*r* = 0.32	*r* = −0.29	*r* = 0.06	*r* = 0.47	*r* = −0.17	*r* = −0.30
	*p* = 0.034			*p* = 0.003		*p* = 0.034

Trunk lean	*r* = 0.09	*r* = 0.26	*r* = 0.19	*r* = 0.27	*r* = −0.02	*r* = −0.22
		*p* = 0.024				

Arm lean	*r* = 0.08	*r* = 0.23	*r* = 0.24	*r* = 0.07	*r* = −0.06	*r* = −0.15

Leg lean	*r* = 0.01	*r* = 0.09	*r* = 0.17	*r* = 0.38	*r* = −0.11	*r* = −0.37
				*p* = 0.015		

TT: total testosterone, cFT: calculated free testosterone, DHEAS: dehydroepiandrosterone sulfate, SHBG: sex hormone binding globulin, IGF-1: insulin-like growth factor 1, HbA1c: glycated hemoglobin, and DXA: dual energy X-ray absorptiometry.
